# Comparing health service usage of migrant groups in Australia: Evidence from the household income and labour dynamics survey of Australia

**DOI:** 10.1016/j.jmh.2024.100277

**Published:** 2024-10-10

**Authors:** Heather Brown, Emily Breislin

**Affiliations:** a Lancaster University, Division of Health Research, Lancaster LA1 4AT, United Kingdom; b Lumanity, Steel City House, West Street, Sheffield S1 2GQ, United Kingdom

**Keywords:** Health service usage, Migrants, Australia, Zero inflated Poisson regression, Non-linear decomposition

## Abstract

**Purpose:**

We explored differences in primary and secondary health care usage across migrants from different regions in Australia.

**Design/methodology/approach:**

Data comes from the Household Income and Labour Dynamics of Australia survey from waves 9, 13, and 17 (2009, 2013, and 2017). Zero inflated Poisson regressions and non-linear decompositions were estimated.

**Findings:**

Younger women from South Asia, Latin America and Eastern and Southern Europe and younger men from Eastern and Southern Europe had lower rates of GP visits compared to the host population. Older African men have higher rates of nights in hospital and younger Eastern and Southern European women, older women from the Rest of Asia, and younger African men and women have lower rates of nights in hospital compared to the host population

**Originality:**

This is the first paper to investigate differences in primary and service usage amongst immigrants across the life course. Our results have important implications for planning of health service resources.

**Practical implications:**

Migrants are a heterogenous group and health policy needs to consider these differences to ensure the effectiveness and efficiency of service provision.

## 1 Introduction

Migration levels are increasing as our world continues to become more connected. In Australia, migrants comprise almost 30 % of the total population (Australian Bureau of Statistics 2020). Migrants are a heterogenous group. There are economic migrants and those who migrate for humanitarian reasons (United Nations 2019). Humanitarian migrants may have experienced traumatic or dangerous events which have the potential to cause a range of health issues, both physical and mental. As migrant numbers are increasing and becoming a larger proportion of the population, it is both an essential human right and necessary for continued economic growth that migrants receive the same high-quality healthcare as the host population. The United Nation's (UN) 2030 Sustainable Development Goals (SDGs) are the first set of goals which highlight the importance migration plays in sustainable development, with over half the goals relevant to migrant wellbeing (Migration Data Portal, 2021).

Migration can provide many benefits to the host country. In Australia, managed labour migration plays an important role in creating a more flexible labour market (Dumont and Liebeg 2014). In the UK, migrants provide a solution to staff shortages and as of 2019, 13.3 % of National Health Service (NHS) workers were not British nationals (Health Foundation 2019). Labour migrants compared to other types of migrants make the biggest contribution to the public purse; on average contributing more through taxes than they cost a host country (Dumont and Liebeg 2014). Migration of all types can impact on the demographic composition of the host and source countries. For the host countries migration can contribute to addressing the aging population problem in high income countries. For source countries, remittance payments can promote economic development, but it may also cause brain drain which will have negative economic consequences (Moody 2006).

Migration is a politically emotive topic. There is a common misconception that migration can put a strain on public services. The impact of migrants on public expenditure is dependent upon the assumptions of the model used and how migrants are defined. As migrants are such a heterogenous group, one cannot make conclusions a priori what the impact of migrants will be on the health system. There are a number of theories which predict on average that migrants especially in the short term, will be healthier than the host population. This implies that they will on average require fewer health care resources. The ‘Healthy Migrant Effect’ (Smith, 2015; Renzaho et al., 2016) predicts that because migrants tend to be younger, fitter, and healthier than the host population they will have lower levels of hospitalisation and death. To qualify as an economic migrant, in some countries, there are health and age criterion for entry. Additionally, migrants from lower income countries may have lived healthier lifestyles with less processed foods, less exposure to pollutants and may have been more physically active. Furthermore, for individuals to migrate, it requires them to be able travel and settle into a new life. Normally, this means that migrants are young and healthy enough for the journey. Overtime, the convergence theory suggests that migrant's health and lifestyle tend to converge to that of the host population because of acculturation (Anikeeva et al. 2010). In further support of the concept that on average migrants are healthier is the ‘Salmon Bias theory.’ This suggests immigrants may return to their original country in later life or in poor health (Namer and Razum 2018). Thus, there impact on the health care system of the host country will be minimal.

The evidence supporting the above theories is mixed and depends upon the migrant group studied. Part of this heterogeneity stems from differences in how countries classify migrants (Namer and Razum 2018). Across the European Union, nationality, country of birth, and citizenship are all used to classify migrants (Rechel et al. 2013). Chiswick et al. (2008) found for Australia that the self-reported health of economic migrants converged to the mean for native Australians, but the health of humanitarian migrants remained poor throughout the study period. This suggests that how migrants are defined and classified is important for understanding their health and what this means for their use and need of health services.

Irrespective of the health status of migrants compared to the host population it is cost-effective that migrants can access healthcare on demand. Bozorgmehr and Razum (2015) found costs of healthcare increased when asylum seekers and refugees were initially unable to access treatments, in comparison to when healthcare was not initially restricted. Graetz et al. (2017) conducted a systematic review of health service usage amongst migrants and host populations across several European countries covering 2009 to 2016 and identified various inequalities. In general, emergency care was utilised more by migrants, however outpatient departments recorded a lower use by migrants. Potentially, this suggests there could be barriers which prevent migrants from accessing non-emergency medical care such as routine check-ups or preventative screenings. Additionally, Trummer et al. (2016) analysing data from several European countries highlighted treating migrant populations’ health issues promptly, out of hospital, is more economically effective than waiting until hospitalisation is required.

Renzaho et al. (2016) identified a gap in public health research on migrant health and their health service usage in Australia. Additionally, in the health and migration literature, there is a lack of evidence, looking at health service usage across the life course and across different migrant groups. Understanding this heterogeneity across the life course as well as across migrant groups is important for the cost-effective targeting of services to those most in need. Differences in primary and secondary service usage by migrant groups can provide clear evidence on which groups may have difficulty in accessing services.

In this paper we aim to explore differences in primary and secondary care across migrants from different countries across the life course. Next, we identify how differences in observed characteristics and unobserved barriers in access such as cultural factors, inequities in access to services, or differences in quality of education for example, may explain any of the differences between migrant groups and the host population primary service usage (Rahimi and Hashemi Nazari, 2021). We solely focus on differences in primary care as higher service usage of secondary care by migrant groups may reflect barriers to access for preventative/ basic care. Overall, our results can be used to help plan the provision of health care resources for all to maximise health, reduce health care costs, and minimise health inequalities.

## 2 Theoretical Implications

We hypothesise that younger immigrants from high income countries are less likely to use both primary and secondary health services as they are likely to be healthier than the host population. Younger economic migrants from lower/middle income countries (LMIC) on average are likely to be as healthy or healthier than the host population. Humanitarian migrants who are more likely to be coming from LMICs are likely to have worse health compared to the host population (Matlin et al. 2018). However, if there are barriers to accessing services then primary service usage may be lower for this group and secondary service usage will be higher. The composition of the migrant population (humanitarian or economic) will determine what the overall association will be for those from LMIC.

For older migrants, we hypothesise that if they have had to engage in low skilled manual work throughout their adult life and have faced barriers to health care services as younger adults, they are likely to be in worse health than the host population. This may mean that older migrants use both more primary and secondary services. Conversely, depending on the nature of barriers to services, after being in Australia for a number of years because of acculturation these barriers may have decreased if migrants better understand the health care system. Acculturation would suggest that service usage for older migrants was similar to that of the host population. In terms of country of origins for migrants, if the majority of migrants from LMIC are low skilled then we may observe increased service usage for both primary and secondary care in older ages. If there is an even mix between high skilled and low skilled migrants or high levels of acculturation than these may cancel each other out so we would observe no significant difference in service usage for migrants from these countries and the host population.

## 3 Methods

We use data from the Household Income and Labour Dynamics of Australia survey from waves 9, 13, and 17 (2009, 2013, and 2017) which contain information on health service usage. The HILDA survey began in 2001 and is a household longitudinal survey administered to approximately 17,000 participants asking individuals about their economic and personal well-being, employment and family life (Melbourne Institute, 2021). Design aspects include a representative sample population, yearly completion by individuals and flexibility to include household changes over time (Watson and Wooden 2012). Some central topics included in each wave are education, employment and relationships. Other topics, such as retirement, health and fertility are included on an alternate yearly basis. The majority of data is obtained via in person interviews. A booster sample of 3117 households was added in 2011. Approximately 62 % of the original sample was part of the survey by wave 18 (Watson and Wooden 2021). The survey has received ethical approval from the Human Research Committee at the University of Melbourne.

### 3.1 Outcome variables

**Fig. 1:**
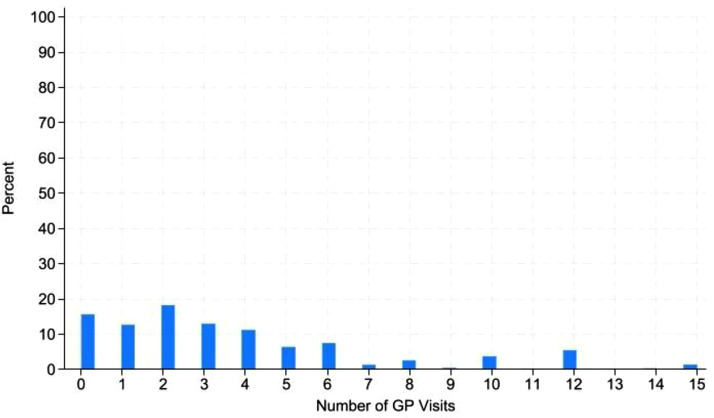
Histogram of Number of GP visits for the whole sample.

**Fig. 2:**
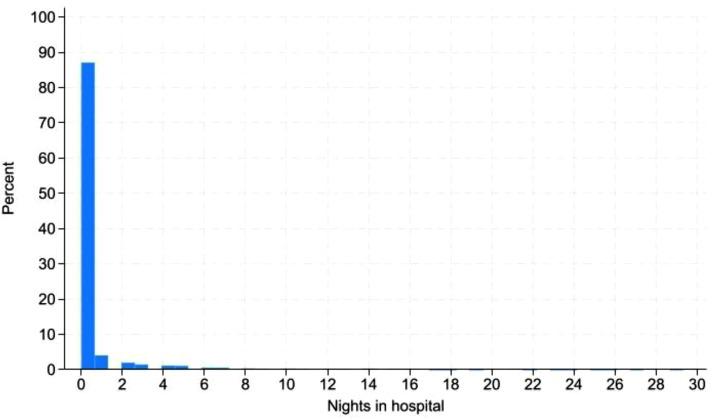
Histogram of Number of Nights in Hospital for the whole sample.

Our first outcome variable is a continuous variable for number of GP visits over the past 12 months. This can include zero. Our second outcome variable is number of nights in hospital over the past 12 months, which can also include zero. We employed a complete-case analysis approach. Thus, we excluded from the analysis any variable that was missing. Using this approach means that for binary and categorical variables the total percentage may not add up to 100 %. We have 40,888 observations for GP visits across the three waves and 40,960 observations for nights in hospital.^1^ Fig. 1, Fig. 2 are histograms of primary and secondary service usage for the full sample. To ease interpretation Fig. 1, Fig. 2 are censored at 15 visits for GPs and 30 visits for nights in hospital (Lesko et al. 2018). We can see that for both outcome variables the distribution is highly skewed with a large number of zeros. This is particularly the case for nights in hospital.

### 3.2 Key explanatory variables

To identify migrants, we use a variable on country of birth of the respondent. We create dummy variables that equal one if the respondent was born in Sub-Sahara or North Africa; South, South East Asia, and Oceania; the rest of Asia; Eastern and Southern Europe; and Central and South America. The base category is if the respondent was born in either Australia or New Zealand. New Zealand is combined with Australia because all New Zealand citizens have no entry barriers to move to Australia. They are eligible to access health care via Medicare and receive some benefits such as family payments (New Zealand Government, 2022) These categories are chosen to reflect the different types of migrants who are likely to come to Australia. Migrants from Africa and Southern Asia are more likely to be humanitarian migrants. Those from Europe, the rest of Asia, and Latin America are more likely to be economic migrants. We exclude migrants from other English speaking countries such as the United Kingdom, Ireland, Canada, and the United States. Because of small numbers we also excluded migrants from other Northern European countries.

In the HILDA survey, age is a continuous variable. However, for ease of interpretation, we divide our sample into two age groups. Those who are between 16 and 50 and those who are older than 51 or older. We choose this grouping as it is likely that as individuals become middle aged (51+) they are more likely to have multiple chronic health conditions which will impact on service usage (Bezerra de Souza et al. 2021). Because of small sample sizes, we could not use a more granular breakdown of age data.

The key determinants of health services usage are based upon individual and contextual factors that are likely to affect access (Andersen and Davidson 2007). The variables included in our analysis were marital status, educational attainment, area level disadvantage, and employment. All equations are estimated separately by gender.

### 3.3 Econometric Framework

To account for the large number of zeros in primary and secondary service usage as can be seen in Fig. 1, Fig. 2, we employ a zero inflated Poisson regression. The model includes both a Poisson and logistic distribution. The model assumes with probability *p,* the only observation is zero and with probability, *1-p,* a Poisson (λ) random variable is observed (Lambert 1992). We assume that health status is a function of the probability of reporting a zero. Those in excellent health are more likely to have zero usage which corresponds with the logit model; however it is also likely that those with worse health may face barriers to health service usage and also have observed zero usage.

Formally the probability distribution for primary and secondary service usage can be presented by (NCSS, 2022):

(1) $$\mathrm{Pr}\left(HS_{i}=j\right)=\left\{ \begin{array}{c} \pi_{i}+\left(1+1\pi_{i}\right)\exp\left(-\beta_{1}X_{i}+\beta_{2}Im_{i}\right)\;if\;j=0\\ 1-\pi_{i}\frac{\mu_{i}^{HS_{i}}\exp\left(-\beta_{1}X_{i}+\beta_{2}Im_{i}\right)\;}{HS_{i}!}\;if\;j=1\; \end{array}\right.$$

Where $\pi_{i}$ is the logistic link function represented by:

(2) $$\pi_{i}=\frac{\beta_{1}X_{i}+\beta_{2}Im_{i}}{1+\beta_{1}X_{i}+\beta_{2}Im_{i}}$$

*HS_i_* represents primary and secondary service usage for individual *i* depending upon which equation is being estimated. The βs are the parameter of coefficients to be estimated. The vector *X* includes individual characteristics related to health service usage and the vector *IM* included the dummy variables for either being an immigrant or country of origin depending upon the model being estimated.

The regression coefficients are estimated by maximum likelihood. Standard errors are clustered to account for multiple observations by some individuals.

We start with a base model of the determinants of service usage which includes only a dummy variable for being an immigrant. Next, we estimate models with the different country of origin region dummies separately. This is because of multicollinearity issues when including all the country of origin dummies in one equation. All models were estimated for younger and older age groups and gender separately. It is likely that zero health service usage will be different by gender and across the age groups.

To understand the influence of inequities in access for primary care, differences in individual characteristics such as educational attainment, and cultural beliefs about health and health service usage we employ a decomposition approach for non-linear models (Sinning et al. 2008). We hypothesis that there are likely to be more barriers for primary care than secondary care which is why we focus on primary care only. The non-linear decomposition approach differs from the original Oaxaca-Blinder decomposition in that instead of deriving the conditional expectations of the means between groups to obtain the differences stemming from observed characteristics, unobserved characteristics, and the interaction of the two; we instead derive the sample counterparts of the conditional expectations. We employ bootstrapping to obtain standard errors and confidence intervals. Similar to a linear Oaxaca decomposition approach the model is sensitive to the reference group choice and regression specification (Sinning et al. 2008). Models are estimated separately comparing migrants from Sub-Sahara or North Africa; South, South East Asia, and Oceania; the rest of Asia; Eastern and Southern Europe; and Central and South America to people born in Australia or New Zealand.

## 4 Results

Table 1 shows descriptive statistics for the entire sample. The mean number of GP visits per year is 4.90 and the mean number of nights in hospital is just under one (0.98). Approximately 60 % of the sample is between the ages of 16–50 and 38 % of the sample is 51 or older. 52 % of the sample is female. 63 % of the sample is employed. Approximately 5 % of the sample originates from the South of Asia, 3 % of the sample emigrated from Eastern and Southern Europe, 2 % emigrated from the Rest of Asia, and 1 % of the sample emigrated from Africa and Latin American. Approximately 81 % of the sample was born in either Australia or New Zealand. 41 % of the sample lives in an area of high deprivation. This suggests that our sample on average lives in a more deprived area than mean of the Australian population. Approximately 25 % of the sample has a university level education or higher.

**Table 1:** Descriptive Statistics.

Variable	Number of Observations	Mean	Std Dev	Min	Max
Number of GP Visits in Past 12 Months	45,235	4.90	6.93	0	170
Number of Nights in Hospital in Past 12 Months	45,203	0.98	6.34	0	365
Age 16–50	45,235	0.60	0.49	0	1
Age 51+	45,235	0.38	0.49	0	1
Female	45,235	0.52	0.50	0	1
Married	45,234	0.62	0.49	0	1
Country of Birth: South Asia	45,221	0.05	0.22	0	1
Country of Birth: Africa	45,221	0.01	0.12	0	1
Country of Birth: Rest of Asia	45,221	0.02	0.15	0	1
Country of Birth: Caribbean/Latin America	45,221	0.01	0.09	0	1
Country of Birth: Eastern and Southern Europe	45,221	0.03	0.17	0	1
Country of Birth: Australian/New Zealand	45,221	0.81	0.39	0	1
Highest 4 Deciles of Relative Socioeconomic Status Advantage/Disadvantage	45,230	0.41	0.49	0	1
Education: Basic Qualifications	45,212	0.15	0.36	0	1
Education: Some higher education	45,212	0.31	0.46	0	1
Education: University	45,212	0.24	0.43	0	1
Employed	45,362	0.63	0.48	0	1

Next, Table 2, Table 3 show mean number of GP visits and nights in hospital by age and country of origin. In Table 2, for adults between 16 and 50, the mean number of GP visits for those born in Australia is 4.22. This number is smaller for those emigrating to Australia ranging from a mean of 4.14 for those from South Asia to 4.18 for those from Latin America. Whereas for adults age 51 or older the mean number of GP appointments is 6.22 for those born in Australia or New Zealand to a mean of 7.21 from those from South Asia to 7.27 for those from Latin America. In Table 3, for nights in hospital the mean number for younger adults between 16 and 50 is 0.62 for those from Australia or New Zealand to a mean of 0.63 for those from South Asia to 0.65 for those from Eastern and Southern Europe. For older adults aged 51 or older, the mean number of nights in hospital for those born in Australia is 1.72 and ranges from a mean of 2.34 for those from South Asia to a mean of 2.45 for those from Africa and Latin America.

**Table 2:** Mean Number of GP visits by age and country of origin.

Region of Birth	Age Group	Number of Observations	Mean	Std Dev	Min	Max
South Asia	16–50	12,472	4.14	6.67	0	170
	51+	50	7.21	7.82	0	150
Africa	16–50	12,013	4.17	6.73	0	170
	51+	7589	7.22	7.82	0	150
Rest of Asia	16–50	12,139	4.17	6.76	0	170
	51+	7665	7.24	7.87	0	150
Caribbean/Latin America	16–50	11,951	4.18	6.73	0	170
	51+	7485	7.27	7.86	0	150
Eastern and Southern Europe	16–50	11,974	4.17	6.72	0	170
	51+	7778	7.24	7.83	0	150
Australian/New Zealand	16–50	25,649	4.22	6.60	0	170
	51+	14,875	6.21	7.46	0	150

**Table 3:** Mean Number of Nights in Hospital by age and country of origin.

Region of Birth	Age Group	Number of Observations	Mean	Std Dev	Min	Max
South Asia	16–50	12,482	0.63	5.15	0	356
	51+	7948	2.34	10.50	0	365
Africa	16–50	12,022	0.64	5.22	0	356
	51+	7615	2.45	10.91	0	365
Rest of Asia	16–50	12,148	0.64	5.20	0	356
	51+	7690	2.40	10.67	0	365
Caribbean/Latin America	16–50	11,960	0.65	5.23	0	356
	51+	7509	2.45	10.79	0	365
Eastern and Southern Europe	16–50	11,983	0.65	5.23	0	356
	51+	7804	2.40	10.66	0	365
Australian/New Zealand	16–50	25,690	0.62	4.60	0	356
	51+	14,906	1.72	8.71	0	365

Table 4 shows the base models estimating the determinants of primary and secondary service usage with only a dummy variable for being an immigrant. Incidence risk ratios are shown. There is a pro-poor usage of GP services, younger men in the most deprived areas have a 1.35 times higher rate of visiting the GP and older men have a 1.28 times higher rate and younger women living in more deprived areas have a rate 1.27 times higher and older women have a rate 1.19 times higher. Men and women across all age groups with a university or postgraduate degree visit the GP less than those with no qualifications. Married men and women across all age groups visit the GP less than those who are single. Employed individuals across all age groups use less GP services. As we predicted, both older immigrant men and women use more GP services than the host population. Younger immigrant women use significantly less GP services than the host population.

**Table 4:** Base Model.

GP Visits					Number of nights in hospital			
	16–50	51+	16–50	51+	16–50	51+	16–50	51+
VARIABLES	Males	Males	Females	Females	Males	Males	Females	Females
Number of kids	1.01	0.99	0.94⁎⁎⁎	1.00	0.98	1.03	0.94*	0.95
	(0.01)	(0.02)	(0.01)	(0.02)	(0.04)	(0.10)	(0.03)	(0.08)
Married	0.98	0.87⁎⁎⁎	0.98	0.83⁎⁎⁎	0.82*	0.78⁎⁎	0.80⁎⁎	0.72⁎⁎⁎
	(0.03)	(0.03)	(0.03)	(0.02)	(0.09)	(0.10)	(0.08)	(0.08)
Basic qualifications	0.83⁎⁎⁎	0.90*	0.94	0.92*	0.89	1.58*	0.81	1.33
	(0.04)	(0.05)	(0.04)	(0.04)	(0.15)	(0.37)	(0.11)	(0.28)
Some higher qualifications	1.00	0.97	0.99	0.92⁎⁎⁎	1.03	0.77⁎⁎	0.90	0.75⁎⁎
	(0.04)	(0.03)	(0.03)	(0.03)	(0.13)	(0.08)	(0.10)	(0.08)
University	0.79⁎⁎⁎	0.80⁎⁎⁎	0.80⁎⁎⁎	0.83⁎⁎⁎	1.14	0.96	0.95	1.01
	(0.03)	(0.03)	(0.03)	(0.03)	(0.20)	(0.17)	(0.12)	(0.15)
Disadvantaged	1.27⁎⁎⁎	1.24⁎⁎⁎	1.20⁎⁎⁎	1.15⁎⁎⁎	1.15	1.03	1.00	0.96
	(0.04)	(0.04)	(0.03)	(0.03)	(0.13)	(0.12)	(0.09)	(0.11)
Employed	0.58⁎⁎⁎	0.60⁎⁎⁎	0.63⁎⁎⁎	0.68⁎⁎⁎	0.54⁎⁎⁎	0.50⁎⁎⁎	0.61⁎⁎⁎	0.55⁎⁎⁎
	(0.02)	(0.02)	(0.02)	(0.02)	(0.06)	(0.05)	(0.05)	(0.06)
Immigrant	1.01	1.07⁎⁎	0.91⁎⁎⁎	1.05*	0.90	1.17	0.94	1.10
	(0.03)	(0.03)	(0.03)	(0.03)	(0.12)	(0.15)	(0.10)	(0.13)
Constant	6.89⁎⁎⁎	7.66⁎⁎⁎	8.36⁎⁎⁎	8.25⁎⁎⁎	7.82⁎⁎⁎	11.09⁎⁎⁎	8.99⁎⁎⁎	12.83⁎⁎⁎
	(0.26)	(0.25)	(0.28)	(0.25)	(1.25)	(1.40)	(1.47)	(1.56)
								
Observations	16,860	8432	17,569	8856	16,881	8445	17,589	8877

Notes: Incidence Risk Ratios are presented. Robust standard errors in parentheses.

⁎⁎⁎ *p* < 0.01,.

⁎⁎ *p* < 0.05,.

⁎ *p* < 0.1.

Next, looking at nights in hospital in Table 4, married men and women across all age groups have a lower rate of spending an additional night in hospital. Employed individuals across all age groups have a lower rate of spending a night in hospital. Older men and women with some higher educational qualifications have a lower rate of spending an additional night in hospital compared to those with no qualifications. Younger men living in the most deprived areas have a 1.28 higher rate of spending an additional night in hospital compared to those living in less deprived areas. None of the immigrant coefficients are statistically significant.

Next in Table 5, Table 6, Table 7, Table 8, Table 9, we look at the results from primary and secondary service usage estimated separately for our four country of origin region dummies: South Asia, Rest of Asia, Africa, Latin America, and Eastern and Southern Europe. Firstly, in Table 5 for South Asia, older men and women have a higher rate of GP usage (1.18 and 1.15 respectively). Younger women have lower rate of GP appointments than the host population. None of the nights in hospital incidence risk ratios are significant. Next, turning to Table 6, older women from the rest of Asia have significantly lower rate of nights in hospital. None of the other incidence risk ratios are significant. In Table 7, for Africa, older African men have a marginally significantly higher rate of GP visits than the host population. Younger men and women from Africa compared to the host population have a significantly lower rate of nights in hospital and older men have a significant higher rate of nights in hospital (2.65). The results with the dummy from Latin America are presented in Table 8. Older men and women compared to the host population have a statistically significant higher rate of GP visits. Younger women have a marginally significant lower rate of GP visits compared to the host population. None of the other incidence risk ratios on secondary service usage are significant. Finally, in Table 9, for Eastern and Southern Europe, younger men and women have statistically significant lower rate of GP visits (although it is only marginally significant for men). Older men and women from Eastern Europe compared to the host population have significantly higher rates of GP visits. Younger women compared to the host population have a significantly lower rate of nights in hospital. Older men from Eastern Europe have marginally significantly lower rate of nights in hospital. Across Table 5, Table 6, Table 7, Table 8, Table 9, the other covariates in the model are similar to the base model.

**Table 5:** South Asia.

	GP visits				Nights in hospital			
	16–50	51+	16–50	51+	16–50	51+	16–50	51+
VARIABLES	Males	Males	Females	Females	Males	Males	Females	Females
Number of kids	1.01	0.99	0.94⁎⁎⁎	0.98	0.98	0.93	0.94*	0.97
	(0.01)	(0.02)	(0.01)	(0.02)	(0.04)	(0.11)	(0.03)	(0.09)
Married	0.98	0.88⁎⁎⁎	0.99	0.83⁎⁎⁎	0.84	0.80	0.80⁎⁎	0.68⁎⁎⁎
	(0.03)	(0.03)	(0.03)	(0.02)	(0.10)	(0.11)	(0.08)	(0.08)
Basic qualifications	0.85⁎⁎⁎	0.82⁎⁎⁎	0.96	0.87⁎⁎⁎	0.92	1.40	0.80	1.17
	(0.04)	(0.05)	(0.04)	(0.05)	(0.17)	(0.39)	(0.11)	(0.27)
Some higher qualifications	1.02	0.96	1.01	0.91⁎⁎⁎	1.07	0.76⁎⁎	0.92	0.73⁎⁎
	(0.04)	(0.03)	(0.03)	(0.03)	(0.14)	(0.09)	(0.10)	(0.09)
University	0.83⁎⁎⁎	0.78⁎⁎⁎	0.82⁎⁎⁎	0.82⁎⁎⁎	1.15	0.94	0.95	1.03
	(0.03)	(0.03)	(0.03)	(0.03)	(0.22)	(0.18)	(0.13)	(0.17)
Disadvantaged	1.29⁎⁎⁎	1.21⁎⁎⁎	1.21⁎⁎⁎	1.12⁎⁎⁎	1.16	1.21	0.99	1.05
	(0.04)	(0.04)	(0.03)	(0.03)	(0.14)	(0.15)	(0.10)	(0.13)
Employed	0.58⁎⁎⁎	0.62⁎⁎⁎	0.63⁎⁎⁎	0.69⁎⁎⁎	0.52⁎⁎⁎	0.57⁎⁎⁎	0.60⁎⁎⁎	0.59⁎⁎⁎
	(0.02)	(0.02)	(0.02)	(0.02)	(0.06)	(0.06)	(0.06)	(0.07)
South Asia	1.00	1.18⁎⁎	0.88⁎⁎⁎	1.12*	0.94	0.96	0.98	0.85
	(0.05)	(0.08)	(0.04)	(0.07)	(0.22)	(0.29)	(0.21)	(0.22)
Constant	6.70⁎⁎⁎	7.74⁎⁎⁎	8.21⁎⁎⁎	8.40⁎⁎⁎	7.72⁎⁎⁎	10.31⁎⁎⁎	9.01⁎⁎⁎	12.78⁎⁎⁎
	(0.26)	(0.26)	(0.28)	(0.27)	(1.30)	(1.40)	(1.55)	(1.62)
								
Observations	15,071	6579	15,723	6935	15,089	6584	15,741	6942

Notes: Incidence Risk Ratios are presented. Robust standard errors in parentheses.

⁎⁎⁎ *p* < 0.01,.

⁎⁎ *p* < 0.05,.

⁎ *p* < 0.1.

**Table 6:** Rest of Asia.

	GP visits				Nights in hospital			
	16–50	51+	16–50	51+	16–50	51+	16–50	51+
VARIABLES	Males	Males	Females	Females	Males	Males	Females	Females
Number of kids	1.02	0.98	0.94⁎⁎⁎	0.99	0.98	0.93	0.94*	0.97
	(0.01)	(0.02)	(0.01)	(0.02)	(0.04)	(0.11)	(0.03)	(0.09)
Married	0.99	0.87⁎⁎⁎	1.00	0.81⁎⁎⁎	0.83	0.82	0.79⁎⁎	0.69⁎⁎⁎
	(0.04)	(0.03)	(0.03)	(0.02)	(0.10)	(0.12)	(0.08)	(0.08)
Basic qualifications	0.85⁎⁎⁎	0.82⁎⁎⁎	0.97	0.84⁎⁎⁎	0.90	1.48	0.76⁎⁎	1.18
	(0.04)	(0.06)	(0.04)	(0.05)	(0.17)	(0.43)	(0.10)	(0.28)
Some higher qualifications	1.03	0.95	1.01	0.92⁎⁎⁎	1.11	0.79*	0.93	0.74⁎⁎
	(0.04)	(0.03)	(0.03)	(0.03)	(0.15)	(0.09)	(0.11)	(0.09)
University	0.82⁎⁎⁎	0.77⁎⁎⁎	0.81⁎⁎⁎	0.81⁎⁎⁎	1.23	0.97	0.98	1.05
	(0.03)	(0.03)	(0.03)	(0.03)	(0.24)	(0.19)	(0.14)	(0.18)
Disadvantaged	1.29⁎⁎⁎	1.19⁎⁎⁎	1.21⁎⁎⁎	1.09⁎⁎⁎	1.12	1.24*	0.97	1.06
	(0.04)	(0.04)	(0.03)	(0.03)	(0.14)	(0.15)	(0.10)	(0.13)
Employed	0.58⁎⁎⁎	0.62⁎⁎⁎	0.62⁎⁎⁎	0.69⁎⁎⁎	0.52⁎⁎⁎	0.57⁎⁎⁎	0.61⁎⁎⁎	0.60⁎⁎⁎
	(0.02)	(0.02)	(0.02)	(0.02)	(0.06)	(0.06)	(0.06)	(0.08)
Rest of Asia	0.92	1.04	0.96	1.05	0.57	1.92	0.88	0.04⁎⁎⁎
	(0.07)	(0.09)	(0.07)	(0.12)	(0.22)	(1.56)	(0.19)	(0.04)
Constant	6.66⁎⁎⁎	7.87⁎⁎⁎	8.18⁎⁎⁎	8.58⁎⁎⁎	7.66⁎⁎⁎	9.83⁎⁎⁎	9.10⁎⁎⁎	12.47⁎⁎⁎
	(0.27)	(0.27)	(0.28)	(0.28)	(1.32)	(1.36)	(1.57)	(1.60)
								
Observations	14,591	6388	15,212	6733	14,608	6392	15,230	6740

Notes: Incidence Risk Ratios are presented. Robust standard errors in parentheses.

⁎⁎⁎ *p* < 0.01,.

⁎⁎ *p* < 0.05,.

⁎ *p* < 0.1.

**Table 7:** Africa.

	GP visits				Number of nights in hospital			
	16–50	51+	16–50	51+	16–50	51+	16–50	51+
VARIABLES	Males	Males	Females	Females	Males	Males	Females	Females
Number of kids	1.01	0.98	0.94⁎⁎⁎	0.99	0.98	1.04	0.94*	0.99
	(0.01)	(0.02)	(0.01)	(0.02)	(0.04)	(0.10)	(0.03)	(0.09)
Married	1.00	0.87⁎⁎⁎	1.00	0.82⁎⁎⁎	0.84	0.84	0.80⁎⁎	0.70⁎⁎⁎
	(0.04)	(0.03)	(0.03)	(0.02)	(0.10)	(0.12)	(0.08)	(0.08)
Basic qualifications	0.86⁎⁎⁎	0.83⁎⁎⁎	0.97	0.85⁎⁎⁎	0.91	1.46	0.75⁎⁎	1.16
	(0.04)	(0.06)	(0.04)	(0.05)	(0.17)	(0.42)	(0.11)	(0.27)
Some higher qualifications	1.04	0.95	1.01	0.92⁎⁎⁎	1.11	0.77⁎⁎	0.93	0.73⁎⁎
	(0.04)	(0.03)	(0.03)	(0.03)	(0.15)	(0.09)	(0.11)	(0.09)
University	0.82⁎⁎⁎	0.77⁎⁎⁎	0.81⁎⁎⁎	0.80⁎⁎⁎	1.22	1.04	0.98	1.05
	(0.03)	(0.03)	(0.03)	(0.03)	(0.24)	(0.20)	(0.14)	(0.18)
Disadvantaged	1.29⁎⁎⁎	1.19⁎⁎⁎	1.21⁎⁎⁎	1.10⁎⁎⁎	1.12	1.20	0.96	1.04
	(0.04)	(0.04)	(0.03)	(0.03)	(0.14)	(0.15)	(0.10)	(0.13)
Employed	0.57⁎⁎⁎	0.62⁎⁎⁎	0.62⁎⁎⁎	0.68⁎⁎⁎	0.51⁎⁎⁎	0.52⁎⁎⁎	0.60⁎⁎⁎	0.59⁎⁎⁎
	(0.02)	(0.02)	(0.02)	(0.02)	(0.06)	(0.06)	(0.06)	(0.07)
Africa	0.94	1.16*	0.88	1.18	0.40⁎⁎⁎	2.64⁎⁎	0.45⁎⁎	1.03
	(0.09)	(0.10)	(0.07)	(0.13)	(0.12)	(1.28)	(0.15)	(0.31)
Constant	6.67⁎⁎⁎	7.89⁎⁎⁎	8.23⁎⁎⁎	8.56⁎⁎⁎	7.62⁎⁎⁎	9.78⁎⁎⁎	9.10⁎⁎⁎	12.49⁎⁎⁎
	(0.27)	(0.27)	(0.29)	(0.28)	(1.32)	(1.35)	(1.59)	(1.60)
								
Observations	14,480	6355	15,045	6680	14,496	6359	15,062	6688

Notes: Incidence Risk Ratios are presented. Robust standard errors in parentheses.

⁎⁎⁎ *p* < 0.01,.

⁎⁎ *p* < 0.05,.

⁎ *p* < 0.1.

**Table 8:** Latin America.

	GP visits				Nights in hospital			
	16–50	51+	16–50	51+	16–50	51+	16–50	51+
VARIABLES	Males	Males	Females	Females	Males	Males	Females	Females
Number of kids	1.01	0.98	0.94⁎⁎⁎	0.99	0.98	0.94	0.94*	0.97
	(0.01)	(0.02)	(0.01)	(0.02)	(0.04)	(0.11)	(0.03)	(0.09)
Married	0.99	0.87⁎⁎⁎	1.00	0.82⁎⁎⁎	0.83	0.82	0.79⁎⁎	0.69⁎⁎⁎
	(0.04)	(0.03)	(0.03)	(0.02)	(0.10)	(0.12)	(0.08)	(0.08)
Basic qualifications	0.86⁎⁎⁎	0.85⁎⁎	0.97	0.86⁎⁎⁎	0.90	1.46	0.75⁎⁎	1.17
	(0.04)	(0.06)	(0.04)	(0.05)	(0.17)	(0.42)	(0.11)	(0.28)
Some higher qualifications	1.04	0.95	1.02	0.92⁎⁎	1.11	0.77⁎⁎	0.93	0.74⁎⁎
	(0.04)	(0.03)	(0.03)	(0.03)	(0.15)	(0.09)	(0.11)	(0.09)
University	0.82⁎⁎⁎	0.77⁎⁎⁎	0.81⁎⁎⁎	0.81⁎⁎⁎	1.24	0.97	0.98	1.05
	(0.03)	(0.03)	(0.03)	(0.03)	(0.24)	(0.19)	(0.14)	(0.18)
Disadvantaged	1.29⁎⁎⁎	1.19⁎⁎⁎	1.21⁎⁎⁎	1.10⁎⁎⁎	1.13	1.24*	0.96	1.06
	(0.04)	(0.04)	(0.03)	(0.03)	(0.14)	(0.15)	(0.10)	(0.13)
Employed	0.57⁎⁎⁎	0.62⁎⁎⁎	0.62⁎⁎⁎	0.68⁎⁎⁎	0.52⁎⁎⁎	0.57⁎⁎⁎	0.60⁎⁎⁎	0.60⁎⁎⁎
	(0.02)	(0.02)	(0.02)	(0.02)	(0.06)	(0.06)	(0.06)	(0.07)
Latin America	0.95	1.34⁎⁎	0.87*	1.56⁎⁎	0.90	0.73	0.72	0.75
	(0.09)	(0.18)	(0.07)	(0.28)	(0.46)	(0.43)	(0.34)	(0.22)
Constant	6.67⁎⁎⁎	7.86⁎⁎⁎	8.22⁎⁎⁎	8.54⁎⁎⁎	7.65⁎⁎⁎	9.91⁎⁎⁎	9.14⁎⁎⁎	12.47⁎⁎⁎
	(0.27)	(0.27)	(0.29)	(0.28)	(1.32)	(1.36)	(1.59)	(1.60)
								
Observations	14,389	6289	14,978	6613	14,406	6293	14,996	6619

Notes: Incidence Risk Ratios are presented. Robust standard errors in parentheses.

⁎⁎⁎ *p* < 0.01,.

⁎⁎ *p* < 0.05,.

⁎ *p* < 0.1.

**Table 9:** Eastern and Southern Europe.

	GP Visits				Nights in Hospital			
	16–50	51+	16–50	51+	16–50	51+	16–50	51+
VARIABLES	Males	Males	Females	Females	Males	Males	Females	Females
Number of kids	1.01	0.98	0.94⁎⁎⁎	0.99	0.98	0.96	0.94*	0.98
	(0.01)	(0.02)	(0.01)	(0.02)	(0.04)	(0.10)	(0.03)	(0.08)
Married	0.99	0.88⁎⁎⁎	1.00	0.83⁎⁎⁎	0.83	0.84	0.80⁎⁎	0.69⁎⁎⁎
	(0.04)	(0.03)	(0.03)	(0.02)	(0.10)	(0.12)	(0.08)	(0.08)
Basic qualifications	0.85⁎⁎⁎	0.89*	0.97	0.89⁎⁎	0.90	1.45	0.75⁎⁎	1.15
	(0.04)	(0.06)	(0.04)	(0.05)	(0.17)	(0.40)	(0.11)	(0.27)
Some higher qualifications	1.03	0.97	1.01	0.94*	1.11	0.77⁎⁎	0.93	0.74⁎⁎
	(0.04)	(0.03)	(0.03)	(0.03)	(0.15)	(0.09)	(0.11)	(0.09)
University	0.82⁎⁎⁎	0.80⁎⁎⁎	0.81⁎⁎⁎	0.81⁎⁎⁎	1.23	0.96	0.98	1.12
	(0.03)	(0.03)	(0.03)	(0.03)	(0.24)	(0.18)	(0.14)	(0.18)
Disadvantaged	1.29⁎⁎⁎	1.21⁎⁎⁎	1.21⁎⁎⁎	1.11⁎⁎⁎	1.12	1.20	0.96	1.07
	(0.04)	(0.04)	(0.03)	(0.03)	(0.14)	(0.15)	(0.10)	(0.13)
Employed	0.57⁎⁎⁎	0.61⁎⁎⁎	0.62⁎⁎⁎	0.68⁎⁎⁎	0.52⁎⁎⁎	0.59⁎⁎⁎	0.60⁎⁎⁎	0.61⁎⁎⁎
	(0.02)	(0.02)	(0.02)	(0.02)	(0.06)	(0.06)	(0.06)	(0.07)
E and S Europe	0.84⁎⁎	1.21⁎⁎	0.81⁎⁎⁎	1.18⁎⁎	0.55	0.69*	0.36⁎⁎⁎	0.71
	(0.07)	(0.09)	(0.07)	(0.08)	(0.21)	(0.15)	(0.10)	(0.19)
Constant	6.73⁎⁎⁎	7.70⁎⁎⁎	8.26⁎⁎⁎	8.34⁎⁎⁎	7.66⁎⁎⁎	9.81⁎⁎⁎	9.12⁎⁎⁎	12.20⁎⁎⁎
	(0.27)	(0.26)	(0.29)	(0.27)	(1.32)	(1.35)	(1.58)	(1.57)
								
Observations	14,458	6566	15,036	6904	14,474	6574	15,053	6914

Notes: Incidence Risk Ratios are presented. Robust standard errors in parentheses.

⁎⁎⁎ *p* < 0.01,.

⁎⁎ *p* < 0.05,.

⁎ *p* < 0.1.

Next in Tables 10, we decompose how much of the difference in GP visits, for those immigrant groups who use less health services stem from observable characteristics and how much stems from unobserved factors such as barriers to access etc. For young women from South Asia, observable characteristics explain 23 % of the difference in GP service usage compared to women from the host population and unobserved factors explain 69 % of the difference. For women from Latin America, there was no significant difference in observable and unobservable characteristic in GP service usage compared to the host population. For men from Eastern and Southern Europe, there was no statistically significant difference in observable characteristics. But unobservable characteristics explain 50 % of the difference in GP service usage. For women from Eastern and Southern Europe, 32 % of the difference in GP service usage compared to the host population can be explained by observable factors and 96 % is explained by unobserved factors. Because of the reference group, the total raw difference for this group is greater than one.

**Table 10:** Decompositions from Younger Women from South Asia, Younger Women from Latin America, Younger Men and Women from Eastern and Southern Europe.

	Characteristics	Unobserved Factors
South Asia (women)	0.23⁎⁎⁎ (0.04)	0.69⁎⁎⁎(0.19)
Latin America (women)	0.21 (0.15)	0.40 (036)
Eastern and Southern Europe (men)	0.11 (0.09)	0.50* (0.27)
Eastern and Southern Europe (men)	0.33⁎⁎⁎(0.08)	0.96⁎⁎⁎(0.25)

Notes: Standard errors are in parenthesis.

⁎⁎⁎ *p* < 0.01, **p < 0.05,.

⁎ *p* < 0.1.

## 5 Discussion

In this paper we explored differences in primary and secondary service by younger (aged 16–50) and older (51+) immigrants from different countries of origin compared to those born in Australia and New Zealand. When we estimate models only with an immigrant dummy, we find that younger women immigrants use less GP services and older immigrants of both genders use more. These results are in line with expectations that younger migrants use less services but older migrants use more. However, when we separate the models by country of origin of migrants, we find that younger women immigrants from South Asia, Latin America, and Eastern and Southern Europe and younger men from Eastern and Southern Europe have lower rates of GP visits compared to the host population. The majority of this difference is explained by differences in coefficients or in other words unobserved characteristics related to factors such as inequity in access, cultural factors, etc. For other migrant groups there was no statistically significant difference in GP service usage. Older African men have higher rates of nights in hospital compared to the host population. Younger Eastern and Southern European women, older women from the Rest of Asia, and younger African men and women have lower rates of nights in hospital.

51 % of Australians believe that immigration is causing an excessive burden on public health services (Ipsos, 2017). Our results show that for migrants from a range of regions and across the life course this unlikely to be the case. There is some evidence that older immigrants have higher rates of GP visits but with the exception of older men from Africa this is not the case for nights in hospital. It is vital that data showing the migrants are not a burden on the health care system is available in the public domain.

The heterogeneity we found across gender, migrant groups and across the life course is consistent with findings from Graetz et al. (2017), who found mixed results of service usage across European countries which may partially be due to differences in classifying migrants and health service systems. This heterogeneity in outcomes across the life course and migrant groups means that our results do not provide strong evidence to support the ‘healthy migrant effect’. Renzaho et al. (2016) draw a similar conclusion that because of heterogeneity in migrant groups one cannot say that there is a healthy migrant effect as some migrants will be healthier than the host population and others especially humanitarian migrants are likely to be in worse physical and mental health.

Our study has a number of strengths and weaknesses. A major strength is the large sample in the HILDA of migrants due to the structure of the Australian population which allows us to compare health service usage between different groups over a number of years. A weakness is that health service usage is self-reported which may lead to recall bias. We also do not know the reason for the health service visit. In addition, because of small sample sizes we could not break down the age categories into smaller groups.

In terms of policy implications, our results highlight that all immigrant groups are different and have different needs so they should not be grouped together. Economic migrants bring many benefits to a country, but long-term planning regarding their health service usage in older ages is needed. For example, deprivation prior to and after migration may increase the risk of morbidity in older age for some migrants. This suggests that promoting greater knowledge/access to preventative services when migrants are young particularly for women migrants may improve health outcomes in older age.

## CRediT authorship contribution statement

**Heather Brown:** Conceptualization, Funding acquisition, Methodology, Writing – review & editing. **Emily Breislin:** Formal analysis, Methodology, Writing – original draft.

## Declaration of competing interest

The authors declare that they have no known competing financial interests or personal relationships that could have appeared to influence the work reported in this paper.

## Footnotes

1 Observations may include data on individuals for each of the three waves or include only one data point for some individuals.
